# The Society of Vascular and Interventional Neurology (SVIN) Mechanical Thrombectomy Registry: Outcomes in Patients With Acute Ischemic Stroke and COVID‐19

**DOI:** 10.1161/SVIN.122.000329

**Published:** 2023-06-14

**Authors:** Ameer E. Hassan, Wondwossen G. Tekle, Sohum K. Desai, Diogo C. Haussen, Mahmoud Mohammaden, Raul G. Nogueira, Sunil A., Sheth, Sergio Salazar‐Marioni, Alexandra Czap, Italo Linfante, Guilherme Dabus, Amy K. Starosciak, Thanh N. Nguyen, Mohamad Abdalkader, Piers Klein, James E. Siegler, Mark Heslin, Lauren Thau, Solomon Oak, Santiago Ortega‐Gutierrez, Mudassir Farooqui, Juan Vivanco‐Suarez, Shahram Majidi, Johanna T. Fifi, Stavros Matsoukas, Weston Gordon, Guillermo Linares, Wilson Rodriguez, Brijesh P. Mehta, Rebecca Sugg, Mohammed Jumaa, David S. Liebeskind

**Affiliations:** ^1^ Valley Baptist Medical Center and University of Texas Rio Grande Valley School of Medicine Harlingen TX; ^2^ Emory University Atlanta GA; ^3^ University of Texas Health Science Center Houston TX; ^4^ Miami Cardiac & Vascular Institute and Miami Neuroscience Institute Baptist Health South Florida Miami FL; ^5^ Boston Medical Center Boston MA; ^6^ Cooper Neurological Institute Cooper University Hospital Camden NJ; ^7^ University of Iowa Hospitals and Clinics IA; ^8^ Mt Sinai NY; ^9^ Saint Louis University MO; ^10^ Memorial Neuroscience Institute Pembroke Pines FL; ^11^ University of South Alabama Mobile AL; ^12^ University of Toledo Toledo OH; ^13^ University of California Los Angeles Los Angeles CA

**Keywords:** acute stroke, COVID‐19, mechanical thrombectomy

## Abstract

**Background:**

Clinical and radiographic outcomes after mechanical thrombectomy in the setting of COVID‐19 infection remain poorly characterized. We sought to determine how COVID‐19 status affects mechanical thrombectomy outcomes in the real‐world setting in the United States.

**Methods:**

The prospectively maintained multicenter mechanical thrombectomy registry from the Society of Vascular and Interventional Neurology was queried for baseline clinical characteristics among patients with and without COVID‐19 who underwent mechanical thrombectomy between March 1 and December 31, 2020 at 12 sites. Primary outcome was the likelihood of good neurological outcomes (90 day modified Rankin scale 0–2) among patients with COVID‐19 treated with endovascular thrombectomy, which was assessed using multivariable logistic regression adjusted for age, National Institutes of Health Stroke Scale, Alberta Stroke Program Early CT Score, and substantial reperfusion (modified Thrombolysis in Cerebral Infarction 2b, 2c, and 3). Secondary outcomes included National Institutes of Health Stroke Scale at 24 hours.

**Results:**

Among 915 patients who underwent mechanical thrombectomy during the study period, 51 patients were positive for COVID‐19 (5.6%). Univariate analysis revealed that compared with patients who were COVID‐19 negative, patients who were positive for COVID‐19 were more likely to be male, nonsmokers, have lower Alberta Stroke Program Early CT Score, and present with intracranial internal carotid artery occlusions (Table 1). They were also less likely to achieve successful reperfusion. Multivariable analysis, however, failed to identify any independent associations with COVID‐19 positive status.

**Conclusion:**

In our cohort, patients postive for COVID‐19 with acute ischemic stroke who undergo mechanical thrombectomy have similar baseline characteristics, imaging features, procedural, and clinical outcomes compared to patients who are negative for COVID‐19 in multivariate analysis. Further analyses are warranted.

**Nonstandard Abbreviations and Acronyms**

ASPECTS: Alberta Stroke Program Early CT Score
LVO: large vessel occlusion

**Clinical Perspectives**

- In this multicenter registry study from centers in the United States, we observed that patients who were COVID‐19 positive with large vessel occlusion were more likely to be male, have lower Alberta Stroke Program Early CT Score, and present with intracranial internal carotid artery occlusions.
- Nonetheless, the overall clinical and procedural outcomes appeared to be similar between patients who were COVID‐19 negative and those who were COVID‐19 positive in the final multivariate analysis.

Severe acute respiratory syndrome coronavirus 2 has become a global pandemic infecting more than 50 million people in the United States and causing more than 800 thousand deaths since January 2020. Its pleiotropic effects include risk of stroke, similar to other respiratory tract infections, through several proposed mechanisms.^1^

As such, we sought to characterize the clinical, periprocedural, radiographic, and clinical outcomes of patients with acute ischemic stroke who were postive for COVID‐19 in the United States using a real‐world multicenter registry.

## Methods

The data are available upon reasonable request to the corresponding author. The prospectively maintained multicenter mechanical thrombectomy registry from the Society of Vascular and Interventional Neurology was queried for baseline clinical characteristics among patients with and without COVID‐19 who underwent mechanical thrombectomy between March 1 and December 31, 2020 at 12 sites. All sites initiated and maintained a locally approved Institutional Review Board‐compliant database allowing for sustainable and prospective real‐time data collection to the Society of Vascular and Interventional Neurology thrombectomy registry. Each participating site performed prospective real‐time data entry utilizing a comprehensive clinical research form that defined mandatory and optional variables utilizing REDCap (Research Electronic Data Capture; Vanderbilt University, TN). Participating centers were instructed to enter procedural data in real‐time for accuracy. Data quality checks were performed to ensure continuous and complete data entry. New data were exported to the coordinating center (Emory University, GA, USA) which checked for quality and merged data if all standards were met. Mandatory variables were expected to have a completion of 98%, with the exception of 90‐day modified Rankin scale with an expected completion rate of >80%. Primary outcome defined as likelihood of good neurological outcomes (90 day modified Rankin scale 0–2) among patients who were COVID‐19 positive and treated with endovascular thrombectomy, which was assessed using multivariable logistic regression adjusted for age, National Institutes of Health Stroke Scale, Alberta Stroke Program Early CT Score, and substantial reperfusion (modified Thrombolysis in Cerebral Infarction 2b, 2c, and 3 [mTICI]). mTICI 2b was defined as greater than or equal to 50% of target vessel territory reperfusing whereas TICI 2c was defined as near complete perfusion except few distal cortical emboli. mTICI 3 was defined as complete reperfusion. Secondary outcomes included National Institutes of Health Stroke Scale at 24 hours.

### Statistical Analysis

After assessing normality of distributions by the Shapiro–Wilk test, continuous variables were reported as median [interquartile range]. Categorical variables were reported as frequencies and percentages. Comparison of continuous was performed using Mann–Whitney U test. Categorical variables were compared using Pearson's chi square (X^2^) or Fisher exact test as appropriate. A backward stepwise regression analysis was performed to find a reduced model that best explains the association of different variables with COVID‐19. Variables with *P*<0.1 in the univariate (sex, race, diabetes, current smokers, stroke pathogenesis, baseline Alberta Stroke Program Early CT Score, site of occlusion, procedure duration, full reperfusion [mTICI3], and parenchymatous hematoma type 2) were added to the model. Variables with *P*>0.10 were excluded from final model. Significance was set at *P* < 0.05, and all *P* values were 2‐sided. Statistical analyses were performed using SPSS 27 software (IBM Armonk, NY, USA).

## Results

A total of 915 patients who underwent mechanical thrombectomy during the study period were included, of which 51 patients were positive for COVID‐19 (5.6%). Median age was 65 years (interquartile range 55–73) within the COVID positive group. Compared with patients who were negative for COVID‐19, patients who were postive for COVID‐19 were more likely to be male, nonsmokers, have lower Alberta Stroke Program Early CT Score, and present with intracranial internal carotid artery or carotid T occlusions (Table 1). While they were also less likely to achieve successful near‐complete or complete reperfusion defined as mTICI 2c/3, this did not appear to affect presence of good outcome at 90 days (*P* = 0.41). Patients who were COVID‐19 positive were not more likely to have intracerebral or parenchymal hemorrhage after thrombectomy (*P* = 0.29 and 0.07, respectively) or increased mortality (*P* = 0.5) compared with their counterparts who were COVID‐19 negative. Multivariable regression analysis (Table 2) however failed to demonstrate any independent variable associated with patients who were COVID‐19 positive who underwent mechanical thrombectomy for large vessel occlusion (LVO).

**Table 1:** Baseline, Procedural Characteristics, and Outcome Among Patients With and Without COVID‐19

	COVID‐19 N=51	Non‐COVID‐19 N = 864	*P* value
Age	N=51 65 [55–73]	N=862 68 [57–79]	0.14
Female	16/51 (31.4)	411/861(47.7)	0.029^*^
Race or ethnicity			
White	25 (49)	440 (50.9)	
Black	10 (19.6)	213 (24.7)	
Hispanic	9 (17.6)	67 (7.8)	0.17
Asian	2 (3.9)	27 (3.1)	
Others	5 (9.8)	117 (13.5)	
Hypertension	37 (72.5)	653 (75.6)	0.63
Diabetes	23 (45.1)	277 (32.1)	0.05
Current smokers	5/51	213 (24.7)	0.017^*^
AF	12 (23.5)	243 (28.1)	0.48
CHF	6 (11.8)	163 (18.9)	0.20
Hyperlipidemia	23 (45.1)	358 (41.4)	0.61
Prior stroke	7 (13.7)	163 (19.1)	0.34
BMI (kg/m^2^)	N=43 43.31 [35.44–49.71]	N=638 44.71 [37.26–53.65]	0.16
Baseline glucose (mg/dL)	N=32 136.5 [113.5–217.25]	N=709 127 [107–158]	0.07
Stroke pathogenesis			
Cardioembolic	25 (49)	367 (42.5)	0.068
ICAD	5 (9.8)	93 (10.8)	
Large vessel atherosclerosis	0 (0)	72 (8.3)	
Arterial dissection	2 (3.9)	13 (3.9)	
Other determined	5 (9.8)	49 (5.7)	
Unknown	14 (27.5)	270 (31.3)	
Baseline NIHSS score	N=51 17 [11–21]	N=860 16 [10–21]	0.79
Baseline ASPECTS	N=39 8 [6–9]	N=685 9 [8–10]	0.008^*^
LKW to puncture	N=44 366.5 [230.75–678.5]	N=790 347 [207–675]	0.77
IV‐tPA	18/49 (36.7)	298/859 (34.7)	0.77
Site of occlusion	N=51	N=857	
Isolated ext. ICA	2 (3.9)	38 (4.4)	0.037^*^
Intracranial‐ICA	11 (21.6)	138 (16.1)	
MCA‐M1	26 (51)	439 (51.2)	
MCA‐M2	3 (5.9)	151 (17.6)	
MCA‐M3	3 (5.9)	9 (1.1)	
ACA	12	4 (0.5)	
Basilar	3 (5.9)	61 (7.1)	
PCA	2 (3.9)	172	
GA	19 (37.3)	319 (37.2)	≥0.99
Procedural duration	N=47 28 [17–50]	N=808 34 [22–54]	0.07
Number of passes	2 [1–3]	1 [1–3]	0.38
Successful reperfusion (mTICI2b‐3)	46 (90.2)	792/858 (92.3)	0.59
Near/full reperfusion (mTICI2c/3)	25 (49)	562/858 (65.5)	0.017^*^
Full reperfusion (mTICI3)	15 (29.4)	443 (51.6)	0.002^*^
SICH	1/50 (2)	46/846 (5.2)	0.29
PH type2	3/50 (6)	15/846 (1.8)	0.07
24 h NIHSS score	N=32	N=637	
	13 [5.25–18.75]	10 [4–18]	0.18
90‐d mRS 0–2	14/45 (31.1)	265/713 (37.2)	0.41
90‐d mRS 0–3	22/45 (48.9)	369/713 (51.8)	0.71
90‐d mortality	15/45 (33.3)	204/713 (28.6)	0.50

ACA indicates anterior cerebral artery; AF, atrial fibrillation; ASPECTS, Alberta Stroke Program Early CT Score; BMI, body mass index; CHF, congestive heart failure; GA, general anesthesia; ICA, internal carotid artery; ICAD, intracranial atherosclerotic disease; IV‐tPA; intravenous tPA; LKW, last known well; MCA, middle cerebral artery; mRs, modified Rankin scale; mTICI, thrombolysis in cerebral infarction; NIHSS, National Institutes of Health Stroke Scale; PCA, posterior cerebral artery; PH, parenchyma hemorrhage; and SICH, symptomatic intracerebral hemorrhage.

^*^ Indicates statistical significance.

**Table 2:** Multivariable Regression Analysis for Factors Associated With COVID‐19

	OR	95% CI	*P* value
Female	1.42	0.43–4.68	0.56
Diabetes	0.49	0.10–2.25	0.36
Smoker	0.55	0.14–2.23	0.41
Baseline glucose	1.00	0.99–1.01	0.24
Baseline ASPECTS	0.70	0.49–1.02	0.06
Procedural duration	0.99	0.97–1.01	0.43
Near/full reperfusion	0.47	0.14–1.60	0.23
90‐d mRS 0–2	0.91	0.58–1.42	0.67
90‐d mortality	0.58	0.06–5.60	0.64

ASPECTS indicates Alberta Stroke Program Early CT Score; mRS, modified Rankin scale; and OR, odds ratio.

## Discussion

The COVID‐19 pandemic challenged the stroke care infrastructure at many levels from pre‐hospital care, cerebrovascular admissions, periprocedural care in the angiography suite, and postoperative care in the intensive care unit.^2^ Previous single center publications reported poorer than expected outcomes among a subset of patients with COVID‐19 who underwent mechanical thrombectomy for LVO.^3^, ^4^

In this multicenter registry study from centers in the United States, we observed that patients who were COVID‐19 positive with LVO were more likely to be male, nonsmokers, have lower Alberta Stroke Program Early CT Score, and present with intracranial internal carotid artery occlusions. They were also less likely to achieve successful reperfusion. Nonetheless, the overall clinical and procedural outcomes appeared to be similar between patients who were COVID‐19 positive and those who were negative in the final multivariate analysis.

There are several hypotheses that could explain our findings. First, early during the pandemic some centers reported lengthened care delays at the prehospital level and between imaging and groin puncture as well as that may have impacted clinical outcomes.^3^ Additionally, we reported that delays in administering IV thrombolysis during the pandemic in patients with acute stroke t were associated with worse outcomes.^5^ As the pandemic has continued, neurointerventionalists particularly within the registry, built on the experience of others and adapted their protocols.^6^ Later analysis revealed no delays in endovascular therapy during the COVID‐19 era with the pre‐COVID‐19 era possibly negating the effect of early experiences among patients with acute LVO who were postive for COVID‐19.^7^, ^8^ Additionally, many centers deferred or delayed elective neurointerventional cases as part of local efforts to free bed capacity including at centers within the registry. This was done preemptively in anticipation of a surge of patients with COVID‐19.^9^ The unintended consequence was greater availability of the neurointerventional call team possibly resulting in shorter time between arrival and angiography for acute LVO, especially among outside transfers.^8^, ^10^

Additionally, the multicenter nature and the multivariate analysis has also muted any regional differences in health care utilization already present prior to pandemic. For example, previous discrepancies among border state Hispanic patients with stroke documented in other papers were not demonstrated in this study with regards to COVID‐19.^11^

## Limitations

This study was a large, multicenter collaborative effort to evaluate the effect of a COVID‐19 on patients with acute ischemic stroke undergoing mechanical thrombectomy; however, it has several limitations. First, despite the large number of patients treated, the sample size of patients who were positive for COVID‐19 was small, underscoring the low frequency of concomitant COVID‐19 infection in patients with LVO in our cohort, as we previously reported.^12^, ^13^, ^14^ Our study is also limited by lack of available data regarding time delay between acquisition of imaging from last known well.

## Conclusions

In our cohort, patients who were positive for COVID‐19 with acute ischemic stroke who underwent mechanical thrombectomy had comparable baseline characteristics, imaging features, procedural, and clinical outcomes compared with patients who were negative for COVID‐19. Further analyses are warranted.

## Sources of Funding

None.

## Disclosures

None.
